# Facile Fabrication of Environmentally-Friendly Hydroxyl-Functionalized Multiwalled Carbon Nanotubes/Soy Oil-Based Polyurethane Nanocomposite Bioplastics with Enhanced Mechanical, Thermal, and Electrical Conductivity Properties

**DOI:** 10.3390/polym11050763

**Published:** 2019-05-01

**Authors:** Xiaogang Luo, Zengcheng Yu, Yixin Cai, Qiangxian Wu, Jian Zeng

**Affiliations:** 1 School of Chemical Engineering and Pharmacy, Wuhan Institute of Technology, Wuhan 430073, Hubei, China; yuzengcheng1505@163.com (Z.Y.); qq408766266@163.com (Y.C.); 2 Green Polymer Laboratory, College of Chemistry, Central China Normal University, Luoyu Road 152, Wuhan 430079, China; 3 Guangdong Provincial Bioengineering Institute (Guangzhou Sugarcane Industry Research Institute), Guangdong Provincial Key Laboratory of Sugarcane Improvement and Biorefinery, Guangzhou 510316, Guangdong, China; jianzeng2008@126.com

**Keywords:** bioplastic, MWCNTs-OH, soy-based polyol

## Abstract

It is challenging to prepare polyurethane bioplastics from renewable resources in a sustainable world. In this work, polyurethane nanocomposite bioplastics are fabricated by blending up to 80 wt % of soy-based polyol and petrochemical polyol with hydroxyl-functionalized multiwalled carbon nanotubes (MWCNTs-OH). The scanning electron microscope (SEM), transmission electron microscope (TEM), and Fourier transform infrared spectroscopy (FTIR) analyses reveal homogeneous dispersion of the MWCNTs-OH in the matrix, as well as interaction or reaction of MWCNTs-OH with the matrix or polymeric methylene diphenyl diisocyanate (pMDI) in forming the organic–inorganic hybrid bioplastic with a three-dimensional (3D) macromolecule network structure. Mechanical properties and electrical conductivity are remarkably enhanced with the increase of the multiwalled carbon nanotube (MWCNTs) loading. Dynamic mechanical analysis (DMA) and thermogravimetric analysis (TGA) results show that the bioplastics with MWCNTs-OH have a better thermal stability compared with the bioplastics without MWCNTs-OH. The composition of the nanocomposites, which defines the characteristics of the material and its thermal and electrical conductivity properties, can be precisely controlled by simply varying the concentration of MWCNTs-OH in the polyol mixture solution.

## 1. Introduction

Nowadays, the requirement of the sustainability of petroleum-based polyurethane (PU) materials has led to increasing demands of the raw materials from natural resources [1]. At the same time, the discard of this type of material into the environment may lead to extremely severe consequences, due to their low biodegradability. For building a more sustainable society, the environmentally-friendly bioplastics produced by renewable resources are in growing demand [2]. Particularly, production of vegetable oil-based polyol has been given the most attention, for both economic and environmental reasons [3]. Soy oil, a renewable bioresource with a relatively low cost and which is abundant in supply, has been used as an alternative raw material to prepare soy-based polyols [4]. Many advantages, such as great biodegradability, low toxicity, and sustainability, are presented, by using these polyols as a reagent for the synthesis of PUs. Soybean phosphate ester polyol (SOPEP) is a great sample [5]—it is made by acid hydrolysis of soybean oil in the presence of phosphoric acid. The SOPEP is suitable for designing rigid biofoams and bioplastics, due to its high functionality and hydroxyl value [6].

Due to the wide applications of PU plastics, PU bioplastics with high efficiency have become a mainstream research field. These PU bioplastics can be formulated by designing properties through the selection of suitable raw materials, while their functionalities can be enhanced by incorporating a variety of fillers into the PU matrix. The incorporation of carbon nanotubes (CNTs) into polymer matrices as a second phase has been extensively studied, in order to produce high-performance polymer nanocomposites [7], thus improving their mechanical, thermal, and electrical properties [8]. Basirjafari et al. studied the effect of CNTs as fillers on the physical and acoustic properties of polyurethane foams [9]. Caglayan et al. analyzed the dispersion of CNTs in polyurethane composites and their effect on the mechanical properties of polyurethanes, indicating that CNTs enhanced the mechanical properties of foams [10]. Tran and Kim found that the blending of CNTs with thermoplastic polyurethane improved its mechanical and electrical properties [11]. Even at a concentration as low as 0.01 wt %, CNTs, used as the electrically conductive nanofillers in plastics, can form PU bioplastics with excellent electrical conductivity [12]. 

In the field of design and the application of vegetable oil-based polyurethane, we have tried our best to make a systematic product design and product application research. The paper is a continuation of our previous work [13,14,15]. The homogenous, finely-dispersed CNTs in the polymer matrix, and the strong interfacial bonding that effects efficient load transfer from the polymer matrix to the CNTs, have become the necessary conditions to take full advantage of the extraordinary properties of CNTs for the reinforcement of the nanocomposites [16,17]. A simple process for preparing nanocomposite bioplastic is provided in this work: blending SOPEP petrochemical polyols with MWCNTs-OH before reacting with polymeric methylene diphenyl diisocyanate (pMDI), after which the compression molding technique is then casted. An industrial method of MWCNT-OH processing is also developed to improve interaction between MWCNTs-OH and the matrix (or pMDI) through covalent integration. 

## 2. Experimental

### 2.1. Materials

SOPEP (functionality = 20) was obtained from Arkema Inc. (Blooming Prairie, MN, United States). Its OH value and acid value are 272 and 35.2 mg KOH/g, respectively, and its chemical structure is shown in our previous work [18]. Jeffol A-630, a petrochemical polyol (OH value = 638 mg KOH/g, functionality = 3.0) and pMDI RUBINATE M (polymeric methyldiphenyl diisocyanate) (NCO% = 31.40, functionality = 2.70), were bought from Huntsman Corporation (Woodlands, TX, USA). MWCNT-OH nanopowders were supplied from U.S. Research Nanomaterials, Inc. (MWCNTs > 95%; OD: 30–50 nm, –OH: 1.06 wt % XPS and Titration). Dabco T-12 (used as a catalyst) was purchased by Air Product, Allentown, PA, USA.

### 2.2. Procedure for Nanocomposites Fabrication

**Figure 1:**
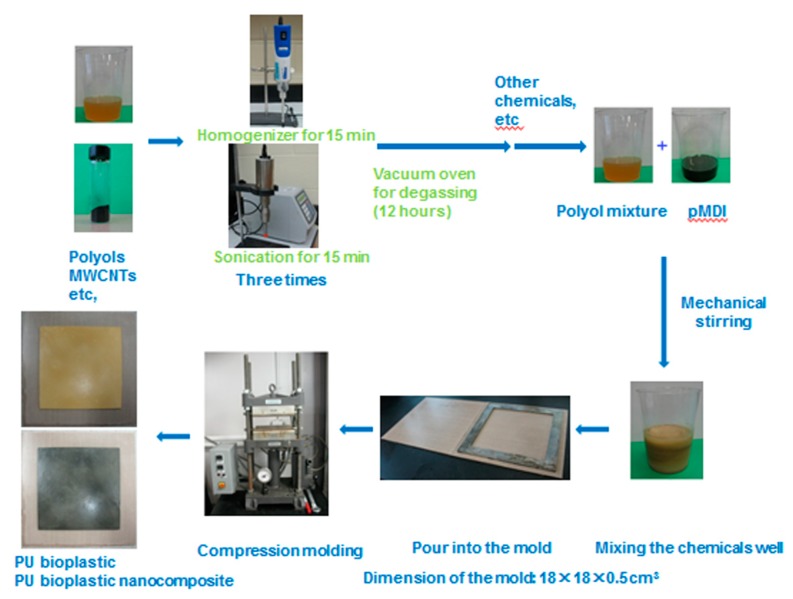
Schematic diagram of preparation of the nanocomposite bioplastics.

The nanocomposites were obtained by a casting compression molding technique [19], from a conventional formulation containing the polyol mixture of SOPEP and Jeffol A-630 (80:20 in weight). With respect to the content of the polyol mixture, the concentrations of all other components were as follows: 1.05 of isocyanate index; 0%, 0.1%, 0.5%, 1.0%, and 2.0% *w*/*w* of MWCNTs-OH; and 0.05% *w*/*w* of catalyst. The schematic diagram of the compression molding preparation process of the nanocomposite bioplastics is illustrated in Figure 1.

In an air-ventilated room, polyols, MWCNTs-OH, and the catalyst were weighed nd added in a beaker. Afterwards, to make sure the MWCNT-OH nanoparticles dispersed well, the above mixture was homogenized and sonicated for 15 min (three times). The pMDI was added to the mixture, immediately mixed for 5 min, and poured into a mold after the well-mixed mixture was degassed for 2 hours under a vacuum condition. Finally, the mold was placed in a Carver Laboratory Press at 120 °C for 1.5 hours. The nanocomposites obtained were designated as BioPUMWCNTs0 (matrix bioplastic), BioPUMWCNTs02, BioPUMWCNTs05, BioPUMWCNTs10, and BioPUMWCNTs20, according to their respective content of MWCNTs-OH.

### 2.3. Characterization

The dispersion and morphology of MWCNT-OH inclusions in the bioplastic systems were assessed with scanning electron microscopy (SEM) (JEOL 6400, Tokyo, Japan) and transmission electron microscopy (TEM) (Philiph, CM10, Amsterdam, The Netherlands). Impact fracture surfaces of the bioplastics were cut into small pieces; to make the surfaces completely conductive, the pieces were sputter-coated with Pt. The dispersion of the MWCNTs-OH in the plastics was measured with TEM.

Fourier transform infrared (FTIR) spectra of the prepared samples were obtained by an FTIR spectrometer (Nicolet 6700, Thermo Nicolet, Madison, WI, United States) equipped with a deuterated triglycine sulphate (DTGS) detector and connected to the software of the OMNIC operating system (Version 7.0 Thermo Nicolet, Madison, WI, USA). 

Densities of these samples were carried out with a model MD-300S in an Alfa Mirage Electronic Densimeter. Tensile and flexural properties were measured with 6–8 samples at 25 °C, according to the standards of ASTM D638 and the ASTM D790. A UMI 43-02 Monitor Impact Tester was used to accomplish the notched Izod impact testing at 25 °C, according to the ASTM D256 standard. 

The dynamic properties of the nanocomposite samples were evaluated by Q800 (dynamic mechanical analysis, DMA TA Instruments); the rectangular sample bars with 50 mm × 12 mm × 2.0 mm were tested in a three-point bend mode at a heating rate of 5 °C/mi, with 1 Hz. Thermogravimetric analysis (TGA) of the samples was carried out under the N_2_, with a TGA Q500 at a heating rate of 10 °C/min from 25 to 600 °C.

Direct current (DC)resistance was measured according to ISO 3915 (four-point method) at a voltage of 500 V, using a megohmmeter (SM-8220, Hioki) for MWCNTs-OH/BioPU samples with high resistance and a multimeter for nanocomposites with low resistance, in combination with a test fixture with 10 mm square copper electrodes [20]. 

## 3. Results and Discussion

### 3.1. Photographs of the Nanocomposite Bioplastics

**Figure 2:**
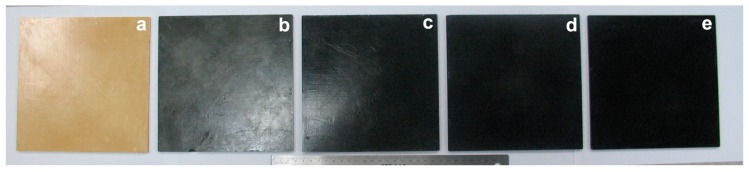
Photographs of the nanocomposite bioplastics: (**a**–**e**) 0.0, 0.1, 0.5, 1.0, and 2.0 wt %, respectively.

The photographs of the nanocomposite bioplastics are shown in Figure 2. All these prepared bioplastics have smooth surfaces. The density, a key feature of polyurethane plastics, greatly affects their properties, such as mechanical and thermal properties, etc. [21,22]; density also determines their applications. These bioplastic samples all have a similar density, between 1.06–1.09 g/cm^3^. The above properties make our products the commercial promising bioplastics in the rigid plastic fields. From the neat BioPU plastic, the color of the nanocomposite bioplastic changes from yellow to black with the addition of MWCNTs-OH, while the morphology of nanocomposite bioplastics changes relatively slightly at varying MWCNT-OH loadings. The above results show that the MWCNTs-OH are uniformly dispersed into the polyols mixture using the homogenization and sonication method. CNTs, especially the MWCNTs-OH, are suitable for being used as reinforcing filler for PU materials designed by direct compression. Similar results have been observed by SEM and TEM analysis.

### 3.2. Dispersion of Hydroxyl-Functionalized Multiwalled Carbon Nanotubes in the Nanocomposite Bioplastics

Dispersion and interaction are two of the critical issues with regard to the processing of MWCNTs-OH/BioPU nanocomposites [23]. The small size and high aspect ratio of MWCNTs-OH lead to the formation of bundles and aggregates. Many techniques, such as high power ultrasonication, surfactant-assisted processing, and functionalization of nanotubes, etc., have been proposed and used to refine the dispersion of CNTs in the matrices [24]. The effective processing of CNTs and polyurethane is still a great challenge, and it determines the properties of MWCNTs-OH/PU nanocomposites.

To get fine dispersion and interactions of the CNTs in industrial-scale production, we adopt a dispersion technique of combining a homogenizer and sonication with commercial, chemically functionalized MWCNTs-OH. 

**Figure 3:**
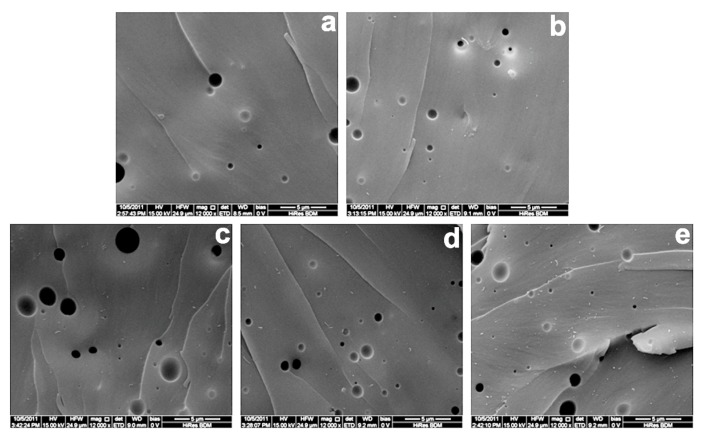
Scanning electron microscope (SEM) images of the nanocomposite bioplastics: (**a**–**e**) 0.0, 0.1, 0.5, 1.0, and 2.0 wt %, respectively.

SEM images of the cross-section display of fractured samples for impact testing of the bioplastics, 0.0, 0.1, 0.50, 1.0, and 2.0 wt % are shown in Figure 3. In these images, the bright spots and lines are attributed to the nanofiller MWCNTs-OH. SEM images show that the MWCNT-OH powders are uniformly distributed throughout the whole matrix without agglomeration, and the minimum fiber pullouts are observed. All the samples have very similar internal structures. Thus, adding MWCNTs-OH also enables the creation of uniform structure nanocomposites, with efficiency improvements in those composites’ mechanical, thermal, and electrical properties. In addition, all these bioplastics have almost the same density. Clearly, good dispersion and random distribution of the MWCNTs-OH in the BioPU matrix was achieved in the preparation of the samples. For all the nanocomposites, the strong van der Waals forces between the BioPU matrix and the MWCNTs-OH exceed the existing inter-tube forces within the primary MWCNT-OH agglomerates, thus allowing fine dispersion during homogenization and sonication to prevent these tubes’ re-agglomeration [25]. This is of great practical importance for the fabrication of CNTs-reinforced polymer composites. Carbon nanotubes can be effectively dispersed via either chemical reaction or hydrogen bonding between the carbon nanotube and polyurethane. The same results are proved by the TEM microphotographs. 

**Figure 4:**
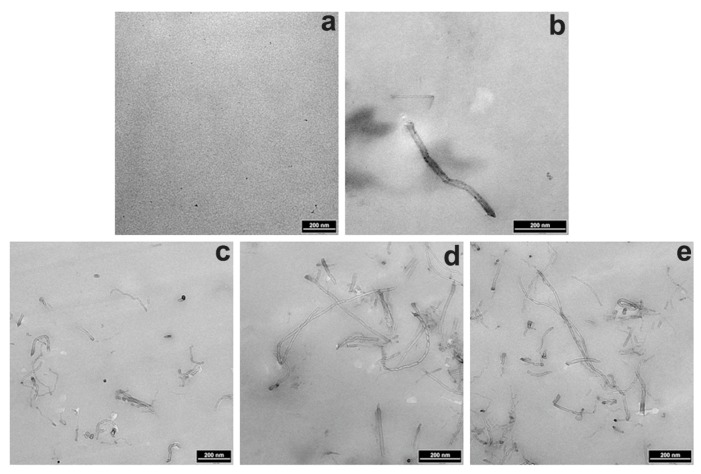
Transmission electron microscope (TEM) images of the nanocomposite bioplastics: (**a**–**e**) 0.0, 0.1, 0.5, 1.0, and 2.0 wt %, respectively.

TEM images of the BioPU nanocomposites 0.0, 0.1, 0.5, 1.0, and 2.0 wt % are shown in Figure 4. MWCNTs-OH in the TEM images appear dark, due to their much higher electron density than the matrix. The remarkable homogeneous distribution of MWCNTs-OH in the BioPU blends can also be clearly seen in these TEM images, which reveal that MWCNTs-OH are uniformly dispersed in the BioPU matrix. At lower loading, the MWCNTs-OH seem to be dispersed as single MWCNTs; with the MWCNT-OH loading increasing, the randomly distributed MWCNTs-OH become closer, and even in the thinly-cut samples present only a two-dimensional (2D) view of the three-dimensional (3D) structure, so the percolative network structure becomes more evident. The MWCNTs-OH are well-dispersed and form a 3D macromolecular network. Good dispersion of the MWCNTs-OH is attributed to the increasing nanotube/polymer matrix interactions, which results from the hydrogen and chemical bonding interaction among the BioPU matrix and the MWCNT-OH powders [26]. The dispersion morphology of MWCNTs-OH in the BioPU matrix also explains the good dispersion of MWCNTs-OH, which is consistent with the development of mechanical, thermal, and electrical conductivity properties of these samples.

### 3.3. Fourier Transform Infrared of the Nanocomposite Bioplastics

The main characteristic absorption bands of urethane moieties—1514, 1710, and 3310 cm^−1^ of NH amide II groups, C=O urethane, and NH, respectively—confirm the structure of the polyurethane.

**Figure 5:**
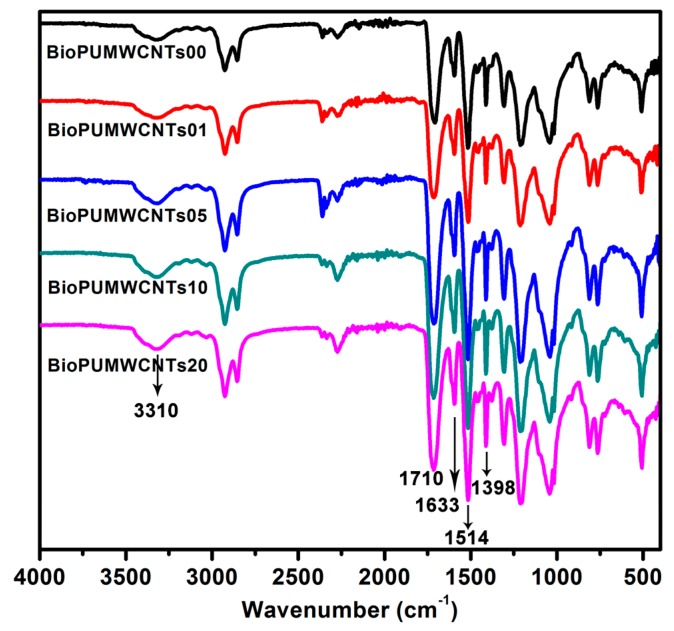
Fourier transform infrared (FTIR) spectra of the nanocomposite bioplastics.

Figure 5 shows that all the bioplastics are prepared successfully. The spectra of these nanocomposite plastics show similar absorption bands as those observed in the native BioPU products. The BioPUs have significant SOPEP content, and are expected to have an increased degree of biodegradability [18]. The highlighted FTIR spectra in 2200–900 cm^−1^ is shown in Figure S1. The appearance of peaks at 1633 and 1398 cm^−1^ are attributed to the C=C bond and bending vibrations of hydroxyl groups on MWCNTs-OH [27]. The stronger intensity of the peak at 1398 cm^−1^ is attributed to the increased content of the MWCNTs-OH [28]. However, the peak can be seen from the FTIR spectra of the nanocomposites, because of the successful introduction of MWCNTs-OH into the matrix BioPU. With the increase in the content of MWCNTs-OH, the intensity of these absorbance bands is also increased; while these absorbance bands are observed at 1753–1709 and 1514 cm^−1^ in the pure BioPU, the peak at 3445 cm^−1^ is attributed to the hydroxyl (–OH) groups on the MWCNTs. The reaction between the hydroxyls on the surface of MWCNTs and pMDI is confirmed by the stronger intensity of two absorption bands at 1633 and 1514 cm^−1^, which are assigned to the absorption of carbonyl groups and NH bending deformation associated with asymmetric stretching, respectively. In practice, a large proportion of the hydroxyl groups on the surface of MWCNTs-OH are likely to react with pMDI [29]. MWCNTs-OH are then covalently bonded to other BioPU chains or pMDI to form the stable 3D molecular polyurethane structure.

### 3.4. Mechanical Properties of Nanocomposite Bioplastics

**Figure 6:**
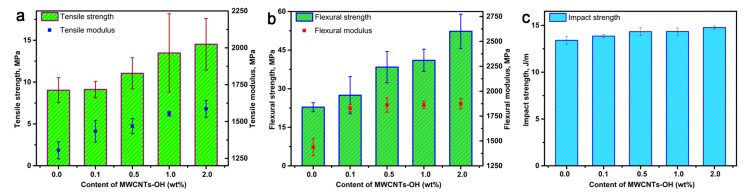
(**a**) Tensile properties, (**b**) flexural properties, and (**c**) impact properties of the nanocomposite bioplastics.

Because of their unique mechanical properties, carbon nanotubes are considered to be the ideal nanofiller candidates for the reinforcement of nanocomposites [30]. The effect of MWCNT-OH reinforcement on the tensile properties of nanocomposite bioplastics is represented in Figure 6a.

Figure 6a shows that with the increase of the loading of MWCNTs-OH from 0.1 to 2.0 wt %, the tensile strength of the nanocomposite bioplastic increases from 9.03 to 14.53 MPa, and the tensile modulus increases from 1304.08 to 1586.01 MPa. This result shows that reinforcing MWCNTs-OH bond strongly to the BioPU matrix, and the external tensile load can be effectively transferred from the BioPU matrix to the MWCNTs-OH. The effect of MWCNT-OH reinforcement on the flexural properties of the nanocomposites is represented in Figure 6b. A similar result can be observed, as with the increasing of MWCNT-OH loading content, in that the flexural strength and modulus were observed to increase from 22.82 to 52.29 MPa and 1438.26 to 1875.55 MPa, respectively. Figure 6c shows the effect of different MWCNT-OH amounts on the notched Izod impact strength of the nanocomposite bioplastics. With the increasing of MWCNT-OH loading content, the strength of reinforced matrix substantially increases [31]. The impact strength of the bioplastics is evaluated to be 13.41, 13.86, 14.33, 14.34, and 14.75 J/m with the MWCNTs-OH addition of 0, 0.1, 0.5, 1.0, and 2.0 wt %, respectively. The impact strength of BioPU bioplastics with different MWCNT-OH loading content improves from neat BioPU bioplastic by 3.23%, 6.41%, 6.48%, and 9.07%, respectively. The cracks propagate freely in the neat BioPU plastic, while in the nanocomposite bioplastics a crack occurs first, and then propagates from the cracked surface along the MWCNTs-OH, followed by MWCNTs-OH pulling out from the matrix BioPU, thus increasing the absorbed energy by MWCNT-OH-reinforced nanocomposite bioplastics during a test of notch impact [32]. The impact strength of the bioplastics is the result of the fracture energies of the BioPU matrix and the energy required to pull out the filler from the BioPU matrix.

The good dispersion (Figure 3 and Figure 4) and strong interfacial bonding (Figure 5) of the MWCNTs-OH in the BioPU samples enables the creation of functional nanocomposite bioplastics with improvements to their mechanical properties, which suggests effective reinforcement with even very low MWCNT-OH loading in the BioPU, just like our previous work has suggested [19].

### 3.5. Dynamic Mechanical Analysis of the Nanocomposite Bioplastics

The effect of the variation of temperature from 0 to 250 °C on the storage modulus of bioplastic samples (Figure S2) is provided in the Supplementary Materials. The results show that the *T*_g_ of bioplastics increases when the MWCNTs-OH increases, which means that the bioplastics reinforced with MWCNTs-OH have achieved a comparatively higher thermal stability. 

### 3.6. Thermogravimetric Analysis of the Nanocomposite Bioplastics

**Figure 7:**
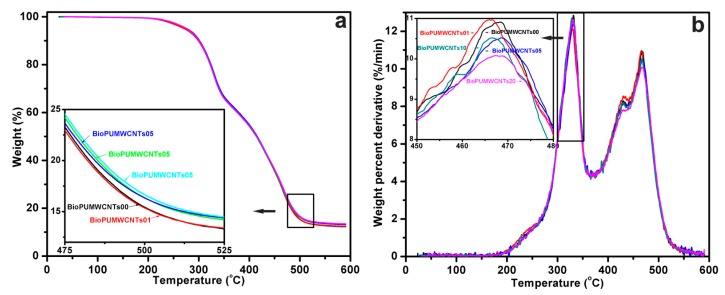
(**a**) Thermogravimetric analysis (TGA) and (**b**) derivative TGA curves recorded under nitrogen at 10 °C/min for the nanocomposite bioplastics

TGA and derivative TGA (DTGA) curves of the nanocomposite bioplastics recorded under N_2_ at 10 °C/min are shown in Figure 7. Basically, the shape of these curves shows no change with or without MWCNT-OH loading (Figure 7a). When the temperature is above 200 °C, the first downturn is observed. Corresponding to neat bioplastic, the DTGA curve (Figure 7b) suggests that there are four main degradation processes revealing four maximum peaks (232, 331, 424 and 468 °C). Similar trends are also observed from the nanocomposite bioplastics, with MWCNT-OH loading of 0.1, 0.5, 1.0, and 2.0 wt %. DTGA curves of MWCNT-OH-reinforced nanocomposite bioplastics are less intense and resolved, showing the same pattern due to the reduction of the volume fraction of the BioPU matrix (Figure 7b). Around 200 °C, urethane bonds start to decompose [33,34], while at the higher temperature the polyol component contributes to degradation [32,35]. These bioplastic samples show almost the same values of the maximum decomposition temperatures (*T*_d_), from 232 to 468 °C, regardless of the MWCNT-OH loading. Soy-based PU might be very similar to petroleum-based PU, as far as hydrolysis is concerned; this is probably due to the weak urethane linkages between –NCO groups in pMDI and the hydroxyl groups in natural triglycerides. Unlike petrochemical polyols that are susceptible to being oxidized to ketones, soy-based polyols consist of more stable paraffinic chains and ester groups [36]. Considering their degradable proportions, the introduction of SOPEP into the nanocomposite leads to a better thermal stability and good degradable property in the material. From the above results, it is concluded that MWCNTs-OH do not affect the basic thermal degradation mechanisms of the bio-based PU, but improve the nanocomposite bioplastics’ thermal stability, which may be attributed to the excellent thermal stability of MWCNTs-OH and their interactions with the BioPU matrix (or pMDI). At the same time, the uniform and fine dispersion of the MWCNTs-OH also improve the thermal stability of the prepared bioplastics. The uniform and fine dispersion of MWCNTs-OH may improve the interfacial adhesion between the MWCNT-OH powders and the BioPU matrix, which can restrict the thermal motion of the BioPU chains and the diffusion of the volatile decomposition products [37].

### 3.7. DC Volume Resistivity of the Nanocomposite Bioplastics

**Figure 8:**
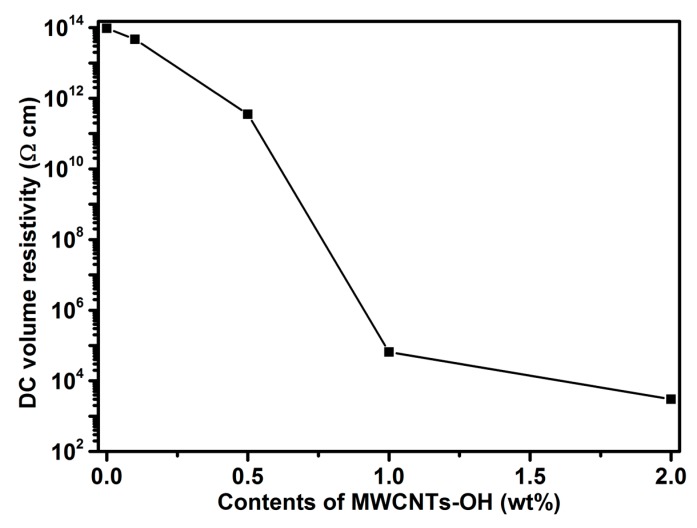
DC volume resistivity of the nanocomposite bioplastics.

The DC volume resistivity of the nanocomposite bioplastics is presented in Figure 8. DC volume resistivity of the materials is decided by the resistivity of both the matrix and the functional fillers [38]. DC volume resistivity of these bioplastics is composition-dependent, and pure BioPU has a resistivity in the order of 10^14^ Ω/cm. With the increase in MWCNTs-OH filler to the added concentration, DC volume resistivities decrease gradually. Compared with the BioPU matrix, MWCNTs-OH have the lower resistivity, and the volume resistivity of the bioplastics regularly decreases with the increasing of the MWCNT-OH load. The electrical conductivity increases about nine orders of magnitude, indicating that the percolation threshold is between a pristine MWCNT-OH weight fraction of 0.5 and 1.0 wt %. When curves align with the percolation theory, the extracted exponent values are small, indicating an effective conductivity increase, possibly because good dispersion of MWCNTs-OH forms a conductive network in the “confined” matrix volume [39].

The DC volume resistivity of the nanocomposites rapidly decreases below 10^3^ Ω cm with the increase in MWCNTs-OH, owing to percolation of the MWCNTs-OH [20]. Moreover, with increasing MWCNT-OH loading, DC resistivity of the bioplastic decreases, because the inorganic particles also contain some moisture on the surface; this helps the ionization of ionic species in the nanocomposite system, thus decreasing the electrical resistivity of these nanocomposite bioplastics [40]. This ultrahigh electrical conductivity of the nanocomposite bioplastic is not due to the degradation of the MWCNTs-OH in nanocomposite bioplastics, but to the moderate preparation condition of the bioplastics, which confirms that the inner walls of MWCNTs-OH are intact during the above high-resolution TEM analysis [20,41].

### 3.8. Schematic Diagram of the Three-Dimensioonal Macromolecular Network Structure Formation of the Nanocomposite Bioplastic

**Figure 9:**
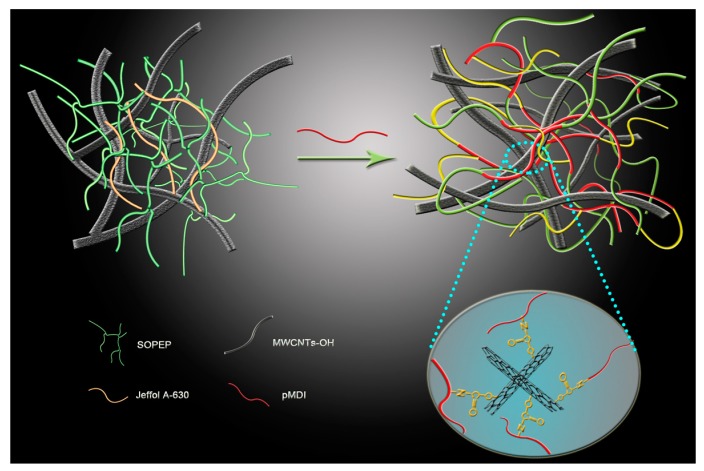
Schematic diagram of the three-dimensional (3D) macromolecular network structure formation of the nanocomposite bioplastic.

Good dispersion, alignment, interfacial stress transfer, and a large aspect ratio are the main four system requirements for effective reinforcement in nanocomposite materials [33]. A schematic diagram of the 3D macromolecular network structure formation of the nanocomposite bioplastic is presented in Figure 9. The material design idea mainly involves (1) a new scalable dispersion process, which has been developed to prepare the nanocomposite bioplastics with appropriate nanofillers dispersion; and (2) because of the multiple hydroxyl groups on the MWCNTs-OH, the prepared nanocomposite bioplastics can form a heavily cross-linked 3D macromolecule structure by covalent bonds among MWCNTs-OH, pMDI, and the BioPU matrix. The MWCNTs-OH become chemically bonded to the 3D macromolecular network structured matrix, and an integral part of the multilayered 3D nanocomposite bioplastics with enhanced mechanical, thermal, and electrical properties, which will enhance the interaction among the MWCNTs-OH powders and the BioPU matrix to improve the MWCNT exfoliation in the bioplastics. The characteristics of the nanocomposite bioplastic and its mechanical, thermal, and electrical conductivity properties can be precisely controlled by simply varying the concentration of MWCNT-OH powders in the polyol mixture solution.

## 4. Conclusions

Soybean phosphate ester polyol (SOPEP) has been chosen properly as a biopolyol to replace 80% petroleum-based polyols, for the design of sustainable, hydroxyl-functionalized, multiwalled carbon nanotubes (MWCNTs-OH)/polyurethane nanocomposite bioplastic by a casting compression molding, with an industrial scale dispersion technique. The dispersion technologies, structure, and usage, as well as the surface and the interface cross-linking of the multiwalled carbon nanotubes (MWCNTs) and their influences on the morphology, chemical structure, mechanical, dynamic mechanical, thermogravimetric properties, and electrical conductivity were investigated. The results reveal fine dispersion as well as strong interaction or reaction of MWCNTs-OH with the matrix or polymeric methylene diphenyl diisocyanate (pMDI) to form the three-dimensional (3D) macromolecular network structure, and there were minor changes in the density of nanocomposite bioplastics. Compared with a neat system, the loading of the MWCNTs-OH in the BioPU matrix resulted in a significant improvement of the mechanical properties, and this was related to the strong MWCNTs-OH–polyurethane interactions. Good dispersion of the MWCNTs-OH powders in the BioPU matrix resulted in the formation of bioPU nanocomposite plastics with better electrical and thermal conductivity than the neat bioPU plastic. This facile fabrication of the bioplastics not only agrees well with the tendency for the materials to be environmentally protective and comprehensive utilization of renewable bioresources, but also fully meets the requirements of advanced performance in industrial electromagnetic interference shielding and deicing materials.

## Supplementary Materials

The supplementary materials are available online at https://www.mdpi.com/2073-4360/11/5/763/s1.
