# Supplementary material for: Facile Fabrication of Environmentally-Friendly Hydroxyl-Functionalized Multiwalled Carbon Nanotubes/Soy Oil-Based Polyurethane Nanocomposite Bioplastics with Enhanced Mechanical, Thermal, and Electrical Conductivity Properties
Source: Polymers (Basel). 2019 May 1;11(5):763. doi: 10.3390/polym11050763 (PMC6572346; doi:10.3390/polym11050763)
Supplement: Supplementary file 1 [file polymers-11-00763-s001.pdf]

Supplementary Information for:

Facile fabrication of environmental friendly MWCNTs-OH/soy oil-based polyurethane nanocomposite bioplastics with enhanced mechanical, thermal and electrical conductivity properties

Xiaogang Luo[1,*], Zengcheng Yu[1], Yixin Cai[1], Qiangxian Wu[2,*], Jian Zeng[3]

[1]School of Chemical Engineering and Pharmacy, Wuhan Institute of Technology, Wuhan 430073, Hubei, China

[2]Green Polymer Laboratory, College of Chemistry, Central China Normal University, Luoyu Road 152, Wuhan 430079, China

[3]Guangdong Provincial Bioengineering Institute (Guangzhou Sugarcane Industry Research Institute), Guangdong Provincial Key Laboratory of Sugarcane Improvement and Biorefinery, Guangzhou, 510316, Guangdong, China

The Supplementary Information contains 4 pages, including 2 Figures

Figures: Fig. S1 and Fig. S2

[*]Corresponding author: School of Chemical Engineering and Pharmacy, Wuhan Institute of Technology, Wuhan 430073, Hubei, China

Tel.: +86-139-86270668;

Email: xgluo0310@hotmail.com; xgluo@wit.edu.cn (X. Luo)

Corresponding author: Qiangxian Wu, Professor, Ph.D.

College of Chemistry, Central China Normal University, Wuhan 430079, China. Tel. and fax: +86-27-67867953;

E-mail: greenpolymerlab@yahoo.com; wuqiangxian@mail.ccnu.edu.cn

FTIR of the nanocomposite bioplastics

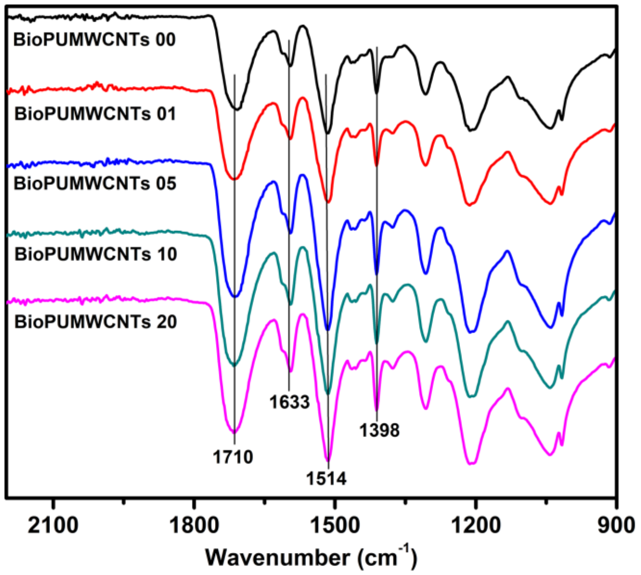

Fig. S1 FTIR spectra (2200-900 cm^{-1}) of the nanocomposite bioplastics

Fig. S1 shows the FTIR spectra in 2200-900 cm^{-1}, and we have marked all the characteristic peaks to clearly seen the difference of peaks. The appearance of peaks at 1633 cm^{-1} and 1398 cm^{-1} are attributed to C=C bond and bending vibrations of hydroxyl groups on MWCNTs-OH [1]. The stronger intensity of peak at 1398 cm^{-1} is attributed to the increased content of the MWCNTs-OH [2]. The reaction between the hydroxyls in surface of MWCNTs and pMDI is confirmed by the stronger intensity of two absorption bands at 1633 and 1514 cm^{-1}, which are assigned to the absorption of carbonyl groups, NH bending deformation associated with asymmetric stretching, respectively.

DMA of the nanocomposite bioplastics

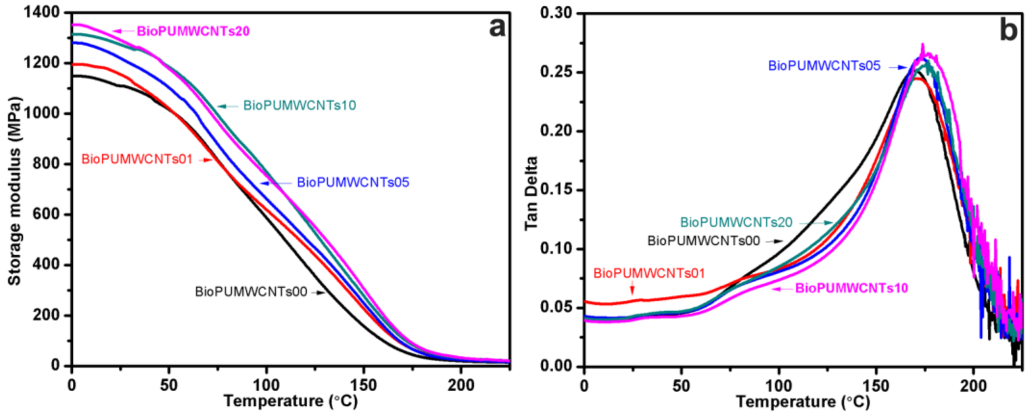

Fig. S2 The temperature dependence of storage modulus (a) and Tan Delta curves (b) of the nanocomposite bioplastics.

Fig. S2 represents the effect of variation of temperature from 0 to 250 °C on the storage modulus of bioplastic samples. The storage modulus of all nanocomposite bioplastics exhibits a downward trend. When the temperatures are below T_g, the modulus values of these nanocomposite bioplastics are higher than that of the native bioplastic. The result supports that the mechanical properties of these rigid nanocomposite bioplastics are improved by the MWCNTs-OH's high mechanical strength and the crosslinking introduced by MWCNTs-OH. The curves of tan delta are shown in Fig. S2b. The temperature values corresponding to the maxima of the peaks are taken as the T_g values of the samples. T_g of prepared bioplastic (170 °C) is shifted to a slightly higher temperature. It is 176 °C when the MWCNTs-OH is loading at 2.00 wt%. The T_g of the bioplastics increases with the MWCNTs-OH

increasing, it means that the bioplastics reinforced with MWCNTs-OH have achieved a comparatively higher thermal stability.

1. Rahimpour, A.; Jahanshahi, M.; Khalili, S.; Mollahosseini, A.; Zirepour, A.; Rajaeian, B. Novel functionalized carbon nanotubes for improving the surface properties and performance of polyethersulfone (PES) membrane. *Desalination* **2012**, *286*, 99-107.

2. Li, S.; Du, X.; Hou, C.; Hao, X.; Jia, J.; Guan, T.; Yi, T.; Ma, G. One-pot two-step perfluoroalkylsilane functionalization of multi-walled carbon nanotubes for polyurethane-based composites. *Composites Science and Technology* **2017**, *143*, 46-55.
